# Unusual Acute Appendicitis Complicated by Sepsis, Evisceration, and Ileostomy: A Case Report

**DOI:** 10.7759/cureus.60360

**Published:** 2024-05-15

**Authors:** Łukasz Stolarski, Tomasz Zawada, Wojciech Tokarczyk, Patryk Patrzałek

**Affiliations:** 1 Intensive Care Unit, District Hospital in Rawicz, Rawicz, POL; 2 Cardiology, Wrocław University Hospital, Wrocław, POL; 3 Surgery, District Hospital in Rawicz, Rawicz, POL

**Keywords:** restoring gastrointestinal continuity, evisceration, severe sepsis, complications, surgery general, acute appendicitis

## Abstract

Acute appendicitis stands as a prevalent cause necessitating surgical intervention globally, predominantly affecting young adults and children, with notably lower incidence among the elderly. Timely diagnosis facilitates effective management, mitigating the risk of severe complications. In this report, we present the case of a 59-year-old patient whose delayed diagnosis and consequently delayed surgical treatment led to serious complications. After the appendectomy, the patient, due to developing sepsis, was transferred to the intensive care unit. On the seventh postoperative day, complications were found in the form of wound dehiscence along with perforation of the jejunum. The second surgery involved a classic laparotomy, encompassing partial resection of the small intestine, and the creation of a single-barrel ileostomy. Further conservative treatment was implemented, and drainage of the abscess was performed. After treatment in the ICU, the patient was transferred to the surgical ward for further treatment. During the hospital stay, further conservative treatment was implemented, resulting in the improvement of the patient's general condition and resolution of symptoms. The patient was discharged home in optimal general condition with recommendations. After six months, the patient was brought to the surgical ward for reconstructive surgery to reestablish gastrointestinal continuity, which was carried out successfully.

## Introduction

The first appendectomy for acute appendicitis is attributed to French physician Mestier, who performed it in 1759 ​[1]​. Acute appendicitis stands as a prevalent reason for surgical intervention on a global scale. The age group with the biggest risk remains young adults and children ​[2]​. 

In typical cases, acute appendicitis begins with gradually increasing pain, which is poorly confined and localized to the central abdominal region. After a varying amount of time, the pain moves to the right iliac fossa, while changing its character to more acute, constant, and well-limited. Many patients also experience a lack of appetite, vomiting, and fever ​[3]​.

Appendicectomy is a relatively safe procedure. The mortality and morbidity are related to the stage of disease and the increase in cases of perforation; mortality after perforation is 5.1 per 1000. The increased mortality and morbidity associated with perforation have been used as justification for high rates of negative appendicectomy, quoted as between 20% and 25% ​[4]​. The most common complications include wound infection, intra-abdominal abscess, and peritonitis ​[5]​. Postoperative results and the chance of developing complications after surgery can be negatively impacted by delaying the therapy of acute appendicitis ​[6]​.

We discuss the case of a 59-year-old patient of Caucasian ethnicity in whom delayed surgical treatment led to severe life-threatening complications. The patient was admitted to the ICU and, after six months, underwent successful surgery to restore the continuity of the digestive tract.

## Case presentation

**Figure 1:**
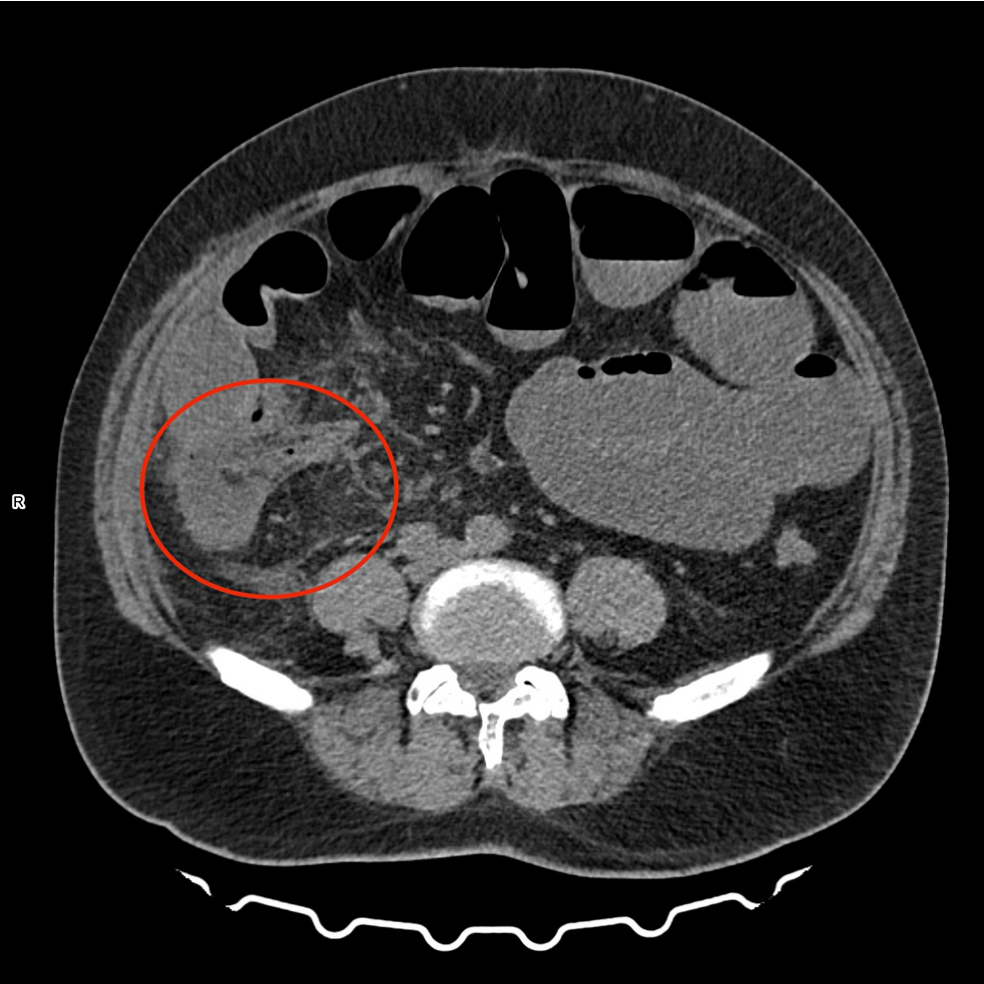
Appendix with inflammatory infiltrate Enlarged appendix to 16 mm (red circle, with fecaliths at its orifice - CT image corresponds to acute appendicitis. Inflammatory infiltration of the surrounding adipose tissue involving the cecum and terminal ileum. Small gas bubbles anterior to the inflamed appendix - likely periappendiceal abscess. Loop of the small intestine, mainly the jejunum, dilated to 52 mm, with a large amount of retained content and fluid levels - features of gastrointestinal obstruction.

A 59-year-old Caucasian with a BMI of 28.1 was admitted to the emergency due to abdominal pain, fever up to 38°C, and weakness persisting for three days. Elevated values of inflammatory markers were observed (Table 1). The patient has not previously undergone any surgery. He gave a history of inflammatory bowel disease without a clear diagnosis. He did not report any other chronic diseases. He also denied previous hospitalizations. The patient presented with tenderness upon palpation at McBurney's point and absent bowel sounds upon auscultation. The abdomen was rigid with muscular guarding, and positive signs of Blumberg's and Rovsing's phenomena were observed. Upon assessment, acute appendicitis with peritonitis was diagnosed based on laboratory, imaging, and clinical findings, necessitating surgical intervention (Figure 1). Under general endotracheal anesthesia, laparotomy was performed, including appendectomy and drainage of a pelvic abscess.

**Table 1:** Key admission parameters

Initial laboratory parameters		Reference range
C-reactive protein (CRP)	332 mg/lL	<5 mg/L
Procalcitonin	11.9 μg/L	<0.1 µg/L
Leukocyte count	15.400/ μL	4000-10 000/μL
Bilirubin	2.41 mg/dL	0.3-1.0 mg/dL
Urea	69.5 mg/dL	15-40 mg/dL
Creatinine	2.2 mg/dL	0.6-1.3 mg/dL

**Figure 2:**
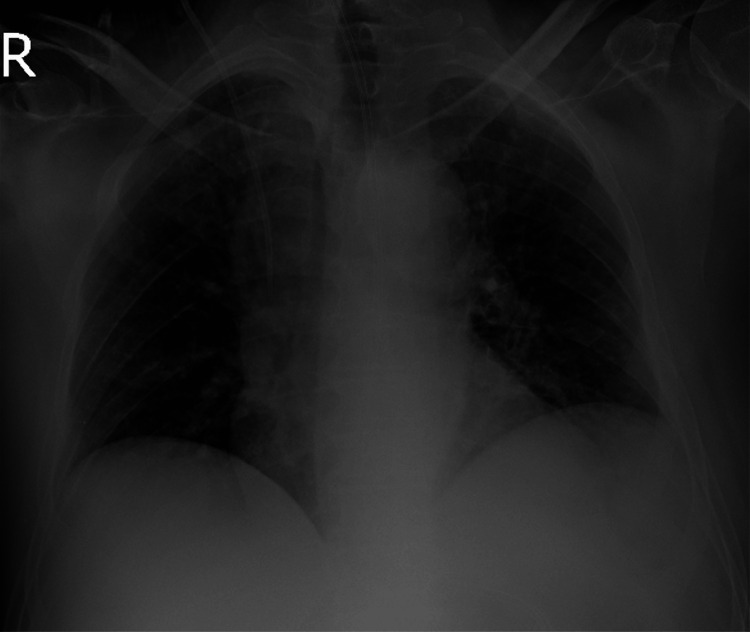
Postoperative X-ray image of the lungs.

**Figure 3:**
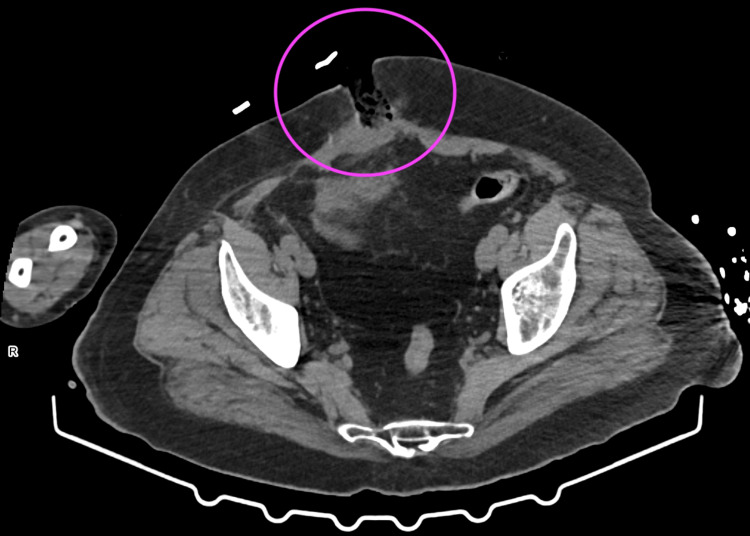
CT image of evisceration Disruption of the continuity of the right rectus abdominis muscle over a length of 25 mm with protruding adipose tissue (pink circle) - hernia with a likely incarcerated loop of intestine with a collapsed lumen.

In the postoperative period, the ￼patient, in a critical condition with developing sepsis, was transferred to the ICU. ￼Auscultation revealed lung crackles, wheezes, and diminished breath sounds, raising suspicion of possible aspiration of intestinal contents. In the radiographic image, inflammatory changes in the lungs were visualized (Figure 2). The Simplified Acute Physiology Score (SAPS) score was 49. Meropenem was initiated as antibiotic therapy. On the seventh postoperative day, complications including evisceration with perforation of the small intestine were identified (Figure 3). Emergency relaparotomy was performed, involving partial resection of the small intestine and the creation of a temporary ileostomy.

The patient remained in critical condition, sedated, febrile, and ventilator-dependent due to *Acinetobacter baumannii* respiratory infection, sensitive to colistin. Linezolid was added to the antibiotic regimen. The patient's condition improved to moderate-severe, with stable consciousness and dependence on vasopressor support and mechanical ventilation. Respiratory support was transitioned from pressure support ventilation (PSV) to volume-controlled synchronized intermittent mandatory ventilation (VC SIMV) due to respiratory decompensation.

After a month of hospitalization in the ICU, the patient's condition significantly improved. Extubation was successful, with good oxygen saturation. The patient was transferred to the surgical ward for further treatment and after a week was discharged in good general condition. Six months later, the patient had been admitted at the surgery ward for reconstructive surgery to restore gastrointestinal continuity. The surgery was successfully performed. After a week, the patient was discharged in good condition.

## Discussion

The creation of the first anatomical drawing of the appendix, attributed to Leonardo da Vinci, dates approximately to 1508 [7]. Berengaria da Carpi is credited with the first description, dating from 1522 [8]. Claudius Amyand's 1735 appendectomy is the earliest known appendectomy. An 11-year-old boy with a fecal fistula and an inguinal hernia was the subject of the case study. After surgery, it was discovered that an appendix was inside the inguinal hernia. The fistula formed because the boy had swollen a pin. These days, an appendix in the inguinal canal is referred to as Amyand's hernia. [9]. The first appendectomy for acute appendicitis is attributed to French physician Mestier, who performed it in 1759 [1].

Acute appendicitis stands as a prevalent reason for surgical intervention on a global scale [2]. Acute appendicitis frequently leads to intense abdominal pain and carries a lifetime risk of 7-16% [3,10]. Regarding gender, the lifetime risk is estimated to range from 8.6% to 12% in men and from 6.7% to 23.1% in women [11,12]. Analyzing age, the most common cases of acute appendicitis are observed in the age range of 10 to 19 years old [2]. The statistical mortality in appendicitis is low and varies between 0.09% in developed health systems and 4% In low-income and middle-income countries [13]. Mortality after perforation is 5.1 per 1000 [4].

The cause of this disease remains poorly understood, and little progress has been made over the past few decades. Although these are more commonly the exception than the rule, direct luminal obstruction can result in appendicitis (usually from a faecolith, lymphoid hyperplasia, or impacted feces; infrequently from an appendiceal or caecal tumor) [13]. Despite reports that several inflammatory factors are associated with appendicitis, such as human cytomegalovirus (HCMV) [14], the full extent of the specific reasons remains unknown [15]. Studies show that despite the lack of identification of a specific gene, people with a positive family history have about a threefold higher risk of developing acute appendicitis [16]. Moreover, according to twin research, around 30% of the variation in the risk of getting appendicitis is due to genetic influences [17].

Obtaining a confident preoperative diagnosis remains a challenge, as the possibility of appendicitis must be considered in every patient with acute abdominal pain. Despite the possibility of using biomarkers and imaging, clinical evaluation still remains the basis for diagnosis [13].

No single inflammatory marker, such as white blood cell count, C-reactive protein (CRP), or other innovative tests like procalcitonin, can accurately diagnose appendicitis with high specificity and sensitivity, but CRP demonstrated a sufficiently high positive likelihood ratio, making it a valuable tool for aiding in the diagnosis of appendicitis. CRP has the potential to be sensitive in identifying appendiceal perforation and the formation of abscesses [18].

Recently, the use of ultrasound alone in the diagnosis of appendicitis has been approached with caution due to its moderate sensitivity (86%) and specificity (81%). Limited availability, especially outside regular hours and weekends, and the need for specialist operators further reduce its utility, although it remains valuable in children due to radiation avoidance needs and thinner musculature [13].

In adolescent and adult populations, computed tomography (CT) has garnered widespread acceptance as the primary imaging modality, utilized in 86% of cases in the United States, demonstrating a sensitivity of 92.3% [19].

The potential use of MRI in acute abdomen cases could mitigate radiation risks in young patients, yet its precise application and accuracy remain understudied. Currently, immediate-access MRI availability is limited globally, and MRI demonstrates comparable accuracy to ultrasound in discerning perforated appendicitis [13].

The Alvarado score is the most commonly reported scoring system for acute appendicitis; it was first described in 1986. However, this score alone lacks the sufficiency to diagnose or exclude appendicitis and requires support from imaging studies [20].

The observed division in the course of the disease suggests that some cases of simple appendicitis may be self-limiting or responsive to antibiotics, while another type often appears to perforate before the patient arrives at the hospital [13]. 

Nowadays, appendectomy procedures are commonly performed on an emergency indication across all surgical departments. The non-specific nature of symptoms and frequently atypical disease progression often pose diagnostic challenges. There is an ongoing discussion regarding the comparison between advocates supporting nonoperative and operative treatments. In the discussed scenario, delayed diagnosis precipitated a perilous course of events for the patient. Complications of appendicitis, such as sepsis and multi-organ failure (MOF) necessitating ICU admission, represent significant challenges globally for healthcare providers and systems. Managing these complications prolongs hospital stays, escalates associated healthcare costs, and impedes the allocation of resources to other patients in need [4]. Although fatalities typically result from complications in older individuals, this threat warrants serious consideration due to the disease's relatively high prevalence.

In acute medical conditions, time is frequently a critical factor, exerting pressure on both patients and healthcare providers. With this in mind, it is imperative to pursue accurate and expeditious diagnoses, commencing with the confirmation or exclusion of the most seemingly significant etiologies. Methodical elicitation of medical history, meticulous physical examination, comprehensive laboratory investigations, and subsequent risk stratification based on these findings should constitute the cornerstone of acute disease diagnosis. It is crucial to recognize that symptoms associated with complications can obfuscate the underlying pathology, thus impeding the diagnostic process. The overarching goal is to safeguard patients from perilous complications and mitigate their attendant sequelae.

## Conclusions

This case demonstrates that a wait-and-see approach in acute appendicitis, while indicated in some instances, should never delay surgical treatment if the patient requires it. Furthermore, it is essential to bear in mind that even seemingly trivial cases of acute appendicitis can pose a serious threat to the life and health of the patient. While delaying appendectomy in cases of suspected uncomplicated appendicitis may appear acceptable, excessive delay in surgical treatment in complicated appendicitis leads to unacceptable complications, exposing healthcare systems to escalating costs at the same time.

## References

[1] Edebohl GM (1899). A review of the history and literature of appendicitis. Medicine in the Americas, 1610-1920.

[2] Wagner M, Tubre DJ, Asensio JA (2018). Evolution and current trends in the management of acute appendicitis. Surg Clin North Am.

[3] Garden OJ, Parks RW (2017). Principles and practice of surgery. 7th ed. Principles and Practice of Surgery. 7th ed.

[4] Blomqvist PG, Andersson RE, Granath F, Lambe MP, Ekbom AR (2001). Mortality after appendectomy in Sweden, 1987-1996. Ann Surg.

[5] Humes DJ, Simpson J (2006). Acute appendicitis. BMJ.

[6] Elniel M, Grainger J, Nevins EJ, Misra N, Skaife P (2018). 72 h is the time critical point to operate in acute appendicitis. J Gastrointest Surg.

[7] Clayton M, Philo R (2014). Leonardo Da Vinci: anatomist. https://www.rct.uk/collection/publications/leonardo-da-vinci-anatomist.

[8] Jackson H, Larkey S, Suden L tum (1934). Jackson’s English translation of Berengarius of Carpi’s “Isagogae Breves”, 1660 and 1664. Isis.

[9] Hutchinson R (1993). Amyand's hernia. J R Soc Med.

[10] Stewart B, Khanduri P, McCord C, Ohene-Yeboah M, Uranues S, Vega Rivera F, Mock C (2014). Global disease burden of conditions requiring emergency surgery. Br J Surg.

[11] Addiss DG, Shaffer N, Fowler BS, Tauxe RV (1990). The epidemiology of appendicitis and appendectomy in the United States. Am J Epidemiol.

[12] Sammalkorpi HE, Mentula P, Leppäniemi A (2014). A new adult appendicitis score improves diagnostic accuracy of acute appendicitis--a prospective study. BMC Gastroenterol.

[13] Bhangu A, Søreide K, Di Saverio S, Assarsson JH, Drake FT (2015). Acute appendicitis: modern understanding of pathogenesis, diagnosis, and management. The. Lancet.

[14] Dzabic M, Boström L, Rahbar A (2008). High prevalence of an active cytomegalovirus infection in the appendix of immunocompetent patients with acute appendicitis. Inflamm Bowel Dis.

[15] Carr N (2000). The pathology of acute appendicitis. Ann Diagn Pathol.

[16] Ergul E (2007). Heredity and familial tendency of acute appendicitis. Scand J Surg.

[17] Sadr Azodi O, Andrén-Sandberg A, Larsson H (2009). Genetic and environmental influences on the risk of acute appendicitis in twins. Br J Surg.

[18] Yu CW, Juan LI, Wu MH, Shen CJ, Wu JY, Lee CC (2013). Systematic review and meta-analysis of the diagnostic accuracy of procalcitonin, C-reactive protein and white blood cell count for suspected acute appendicitis. Br J Surg.

[19] Cuschieri J, Florence M, Flum DR (2008). Negative appendectomy and imaging accuracy in the Washington State Surgical Care and Outcomes Assessment Program. Ann Surg.

[20] Petroianu A (2012). Diagnosis of acute appendicitis. Int J Surg.

